# Enhancement of Dissolution Rate of Indomethacin: Using Liquisolid Compacts

**Published:** 2011

**Authors:** Majid Saeedi, Jafar Akbari, Katayoun Morteza-Semnani, Reza Enayati-Fard, Shirin Sar-Reshteh-dar, Ala Soleymani

**Affiliations:** a *Department of Pharmaceutics, Faculty of Pharmacy, Mazandaran University of Medical Sciences, Sari, Iran.*; b *Pharmaceutical Sciences Research Center, Mazandaran University of Medical Sciences, Sari, Iran.*; c *Department of Medicinal Chemistry, Faculty of Pharmacy, Mazandaran University of Medical Sciences, Sari, Iran.*

**Keywords:** Indomethacin, Liquisolid compacts, Dissolution rate, PEG 200, Glycerin

## Abstract

The potential of liquisolid systems to improve the dissolution properties of a water-insoluble agent (indomethacin) was the purpose of this survey. In this study, different formulations of liquisolid tablets using different co-solvents (non-volatile solvents) were prepared and the effect of several amounts of them on the dissolution behaviour of indomethacin was investigated. It is worth mentioning that the ratio of microcrystalline cellulose (carrier) to silica (coating powder material) was 20 in all formulations. To evaluate any interaction between indomethacin and the other components in liquisolid formulations, the differential scanning calorimeter (DSC) was used. The results showed that the liquisolid formulations exhibited significantly higher drug dissolution rates in comparison with directly compressed tablet. The enhanced rate of indomethacin dissolution derived from liquisolid tablets was probably due to an increase in wetting properties and surface area of drug particles available for dissolution. Moreover, it was indicated that the fraction of molecularly dispersed drug (*F*_M_) in the liquid medication of liquisolid systems was directly proportional to their indomethacin dissolution rate (*D*_R_). An attempt was made to correlate the percentage drug dissolved in 10 min with the solubility of indomethacin in PEG 200 and glycerin. In conclusion, the liquisolid compacts technique can be a promising alternative for the formulation of water insoluble drugs, such as indomethacin into rapid release tablets.

## Introduction

For poorly soluble, highly permeable (Class II) drugs, the rate of oral absorption is often controlled by the dissolution rate in the gastrointestinal tract (1). Therefore, together with the permeability, the solubility and dissolution behaviour of a drug are key determinants of its oral bioavailability (2). Over the years, various techniques have been employed to enhance the dissolution profile and, in turn, the absorption efficiency and bioavailability of water-insoluble salts and polymorphic forms, the formation of water-soluble molecular complexes, the drug micronization, the solid dispersion, the co-precipitation, the liophylization, the microencapsulation, and the inclusion of drug solutions or liquid drugs into soft gelatin capsules are some of the major formulation tools which have been shown to enhance the dissolution characteristics of water-insoluble drugs, however, among them, the technique of “liquisolid compacts” is one of the most promising techniques (3-8).

Formulating liquid medications into solid compacts has been the object of many studies. Jarowski (9, 10), Spireas *et al. *(11, 12, 13), and Javadzadeh *et al. *(7, 14, 15, 16, 17) worked on producing solid solutions and liquisolid based on the concept of the blending liquid medications with selected powder excipients to produce free flowing, readily compressible powders.

The liquisolid compacts are acceptably flowing and compressible powdered forms of liquid medications. The term “liquid medication” implies the oily liquid drugs and solutions or the suspensions of water insoluble solid drugs carried in suitable non-volatile solvent systems termed the liquid vehicles. Using this formulation technique, a liquid medication may be converted into a dry-looking, non-adherent, free flowing and readily compressible powder through a simple blending with selected powder excipients referred to as the carrier and coating materials (11, 12). 

The anti-inflammatory agent indomethacin exhibits poor solubility. This undesirable physical property may increase the incidence of irritating side effects on the GI tract due to a prolong contact time with the mucosa (18). Numerous attempts (19-21) have been made to improve the dissolution rate of this widely used antirheumatic agent, with the purpose of obtaining a more rapid and complete absorption. This can be an ideal candidate for testing the potential of rapid-release liquisolid compacts. Nokhodchi and Javadzadeh studies (3, 15) showed that indomethacin liquisolid compacts which prepared using by Tween 80, propylene glycol and PEG 400, demonstrated a considerably higher drug dissolution rate than those of conventional capsules and directly compressed tablets.

In this study the effects of types of vehicle on the dissolution rate, and the released kinetics were investigated. These effects were analyzed statistically in the same ratios of two nonvolatile solvents with different drug solubility.

## Experimental

*Materials*

Indomethacin was provided by Rouz Daru Co. (Iran). Microcrystalline cellulose (Avicel PH101, FMC Biopolymer, Ireland), nm-sized amorphous silicon dioxide (Mingtai chemical, Taiwan), and polyethylene glycol 200 (PEG 200), glycerin, starch, potassium hydrogen phosphate, sodium hydroxide and magnesium stearate, (Merck, Germany) were used. 

*The preparation of conventional tablet and liquisolid compacts*

Indomethacin conventional tablets were produced by mixing the drug with avicel-silica (ratio of microcrystalline cellulose: silica was 20:1) for a period of 10 min in a cubic mixer (Erweka, Germany). The mixture then mixed with starch (10%) as disintegrating agent for 10 min, and then the Mg stearate (1%) was mixed for 5 min. The mixture was compressed on a 10 mm punch and die using a single punch tableting machine (Korsch, Germany). Sufficient compression was applied in order to produce tablets hardness of 6-7 kp, as determined through using a hardness tester (Erweka, TBH30MD, Germany). This formulation was denoted as DCT and each tablet contains 25 mg of indomethacin, 150 mg of coarse granular microcrystalline cellulose, and 7.5 mg of nm-sized silica.

Several liquisolid of indomethacin compacts (denoted as LS-1 to LS-14) were prepared as follows: Indomethacin was dispersed in PEG 200 or glycerin (these vehicles were used as the liquid vehicle to prepare the liquid medication of the different drug concentrations) with different ratios ranging from 55:45 to 60:70 (drug: PEG200/ glycerin). Then, binary mixtures of microcrystalline cellulose-silica (microcrystalline cellulose as the carrier powder and silica as the coating material with a ratio of 20 R) were added to the mixture containing the drug and PEG 200 or glycerin under continuous mixing in a mortar. Depending on the ratio of the drug and the liquid vehicle in the liquid medication used, different liquid load factor (the liquid load factor, *L*_f_, is the weight ratio of the liquid medication and carrier powder in the liquisolid formulations.) were employed in our liquisolid preparations. These amounts of the carrier and coating materials were enough to maintain acceptable flow and compression properties. Starch (10% w/w) as disintegrant and Mg stearates as lubricant were mixed with all formulations for a period of 10 min and 5 min, respectively. The final mixture was compressed using a single punch tableting machine (Korsch, Germany). The important formulation characteristics of the prepared indomethacin liquisolid compacts are shown in Table 1.

**Table 1:** The key formulation characteristics of the prepared indomethacin liquisolid compacts

**Liquisolid system**	**Liquid vehicle**	**Drug con. in liquid medication (% w/w) (** ***C***_d_ **)**	**Liquid load factor (** ***L***_f_ **)**	**Unit dose (mg)**	**Molecular fraction (** ***F***_M_ **)**
**LS-1**	PEG 200	55	0.278	241.2	0.162
**LS-2**	PEG 200	50	0.250	288.9	0.178
**LS-3**	PEG 200	45	0.227	346.8	0.198
**LS-4**	PEG 200	40	0.208	419.4	0.223
**LS-5**	PEG 200	35	0.192	512.5	0.255
**LS-6**	PEG 200	30	0.178	636.9	0.298
**LS-7**	Glycerin	55	0.370	193.5	0.184
**LS-8**	Glycerin	50	0.333	230.5	0.203
**LS-9**	Glycerin	45	0.303	278.5	0.225
**LS-10**	Glycerin	40	0.278	331.8	0.254
**LS-11**	Glycerin	35	0.256	404.2	0.290
**LS-12**	Glycerin	30	0.238	500.8	0.338
**DCT** ^b^	-	-	-	202.7	0.000

*Spectrophotometeric analysis*

The spectrophotometeric analysis of all indomethacin samples in phosphate buffer (pH 7.2) was performed at 318 nm (UV/Visible spectrophotometer, Varian, Australia). The standard curve was constructed by serially diluting stock solution of the drug at pH 7.2 to obtain concentrations in the range of 5-60 μg/mL using phosphate buffer 7.2 as the diluent. Each sample was analyzed in three days (intraday analyzing) as triplicate.

*Solubility studies*

Solubility measurements were performed according to the method of Higuchi and Connors (22). In brief, solubility studies of indomethacin were carried out in buffer solution pH 7.2, PEG 200, and glycerin. Saturated solutions were prepared by adding excess drug to the vehicles and shaking on the shaker (Memmert, Germany) for 48 h at 25 ± 0.5°C under constant vibration. After this period the solutions were filtered, diluted and analyzed by UV-spectrophotometer. Three determinations were carried out for each sample to calculate the solubility of indomethacin. The solubility of indomethacin in acidic buffer (pH 1.2) was determined as well. These data showed low solubility of drug.

*Dissolution studies*

The USP basket method (Erweka, TD80, Germany) was used for all the *in-vitro *dissolution studies. The dissolution was studied in acidic buffer (pH 1.2) at first. The dissolution data showed low release (less than 6.2%), thus in this method, phosphate buffer pH 7.2 was used as a dissolution media. The rate of stirring was 100 ± 2 rpm. The amount of indomethacin was 25 mg in all formulations. The dosage forms were placed in 900 mL of phosphate buffer and maintained at 37 ± 0.1°C. At appropriate intervals (5, 10, 15, 20, and 30 min) 5 mL of the sample were taken and purified through the filter. The dissolution media was then replaced by 5 mL of fresh dissolution fluid to maintain a constant volume. The samples were then analyzed at 318 nm by UV/Visible spectrophotometer. The mean of at least four determinations was used to calculate the drug release from each of the formulations. 

For assessment and comparison, drug dissolution rates (*D*_R_) were used. To this end, the amount of the dissolved drug (in μg) per minute during the first 10 min, was calculated as follows: 

*D*_R_ *= (M × D)/1000*

Where *M *is the total amount of indomethacin in each tablet (in this study it is 25000 μg) and *D *denotes percentage of the dissolved drug during the first 10 min. 

*Differential scanning calorimetery*

Thermograms of samples (indomethacin, excipients, physical mixture of directly compressed tablets and liquisolid formulations: LS-2 and LS-8) were recorded on a DSC-60 (Perkin elmer). Samples (3-5 mg accurately weighed to 0.01 mg) were placed in aluminum pans and the lids were crimped using a Perkin elmer crimper. Thermal behaviour of the samples was investigated at a scanning rate of 10°C min^-1^, covering a temperature range of 30 -300°C. The instrument was calibrated with an indium standard. 

*Statistical analysis*

All the data were statistically analyzed by analysis of variance followed by Tukey’s multiple comparison tests. A linear regression analysis was used to test associations between two parameters. Results are quoted as significant where p < 0.05. 

## Results and Discussion

Indomethacin was selected as the model drug for this study, since it is a slightly water-soluble substance and, thus, an ideal candidate for testing the potential of rapid-release liquisolid compacts. In addition, it can be easily assayed and quantitated in solution using spectrophotometeric principles and procedures. Beer’s law was obeyed through all the standard curves of our indomethacin solutions which were linear in the concentration range tested. The results of solubility measurements are presented in Table 2. The solubility of indomethacin (pKa = 4.5) in pH 7.2 buffer solution medium at 25°C was found to be 734 μg/mL therefore, according to the USP solubility definition, indomethacin can be considered as a slightly soluble drug at pH 7.2. The results in Table 2 show that the solubility of indomethacin is considerably increased in presence of PEG 200 and glycerin. The increase in the solubility of indomethacin at 25°C in PEG 200 and glycerin was about 121.7 and 138.3 fold respectively compared to pure indomethacin at pH 7.2. 

**Table 2:** The **s**olubility of indomethacin in various solvents

**Solvent **	**Solubility (g/1000 g) **
**Phosphate buffer (pH 7.2) **	0.734
**Polyethylene glycol 200 **	89.3
**Glycerin **	101.5

**Figure 1:**
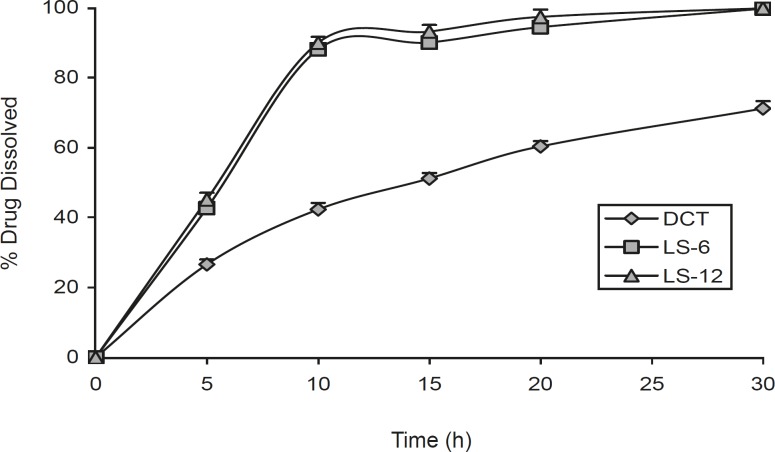
The dissolution profiles of indomethacin liquisolid compacts (LS-6 and LS-12) and directly compressed tablet (DCT). Error bars are standard deviations for at least 4 determinations

Figure 1 shows the dissolution profiles of indomethacin from the liquisolid compacts (LS-6 and LS-12, ratio of drug to vehicle is 3:7) and direct compressed tablet. This Figure shows that the liquisolid compacts produce a higher dissolution rate in comparison with direct compression tablet (p < 0.000) and there was no difference between release rate in LS-6 and LS-12 after 10 and 20 min dissolution study (p = 0.302 and p = 0.089 respectively). Such enhanced drug dissolution rate may be mainly attributed to the fact that this poorly-water-soluble drug is already in solution in PEG 200 and glycerin, while at the same time, it is carried by the powder particles (microcrystalline cellulose-silica) of the liquisolid vehicle. Thus, its release is accelerated due to its markedly increased wetability and surface availability to the dissolution medium. 

**Figure 2:**
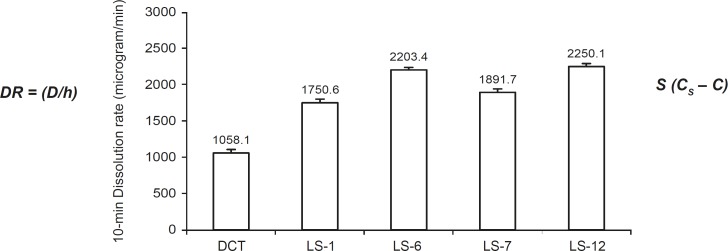
The comparison of the 10 min dissolution rate of indomethacin exhibited by liquisolid compacts containing PEG 200 (LS- 1 and LS-6) and glycerin (LS-7 and LS-12) and directly compressed tablet (DCT). Error bars are standard deviations for at least 4 determinations

Figure 2 shows the *D*_R_ of indomethacin from investigated liquisolid compacts (the highest and the lowest concentrations of PEG 200 and glycerin in formulations) and directly compressed the tablet in the first 10 min. As it is clear from this figure, the liquisolid tablets displayed better *in-vitro *release characteristics than those of the directly compressed tablet (p < 0.001). According to the classic dissolution equation (23): 

*D*_R_ *= (D/h) S (C*_S_ *– C)*

The drug dissolution rate (*D*_R_) of a drug is directly proportional to its concentration gradient *(C*_S_* – C) *in the stagnant diffusion layer and its surface (*S*) available for dissolution. *C*_S_ is the saturation solubility of the drug in the dissolution medium and thus, it is a constant characteristic property related to the drug and dissolving liquid involved. Since all of the dissolution tests for formulations were done at a constant rotational paddle speed (100 rpm) and identical dissolving media, we can assume that the thickness (*h*) of the stagnant diffusion layer and the diffusion coefficient (*D*) of the drug molecules remain almost identical. Therefore, the observed higher dissolution rates of indomethacin from liquisolid tablets are due to the significantly increased surface of the molecularly dispersed indomethacin (12). 

**Figure 3:**
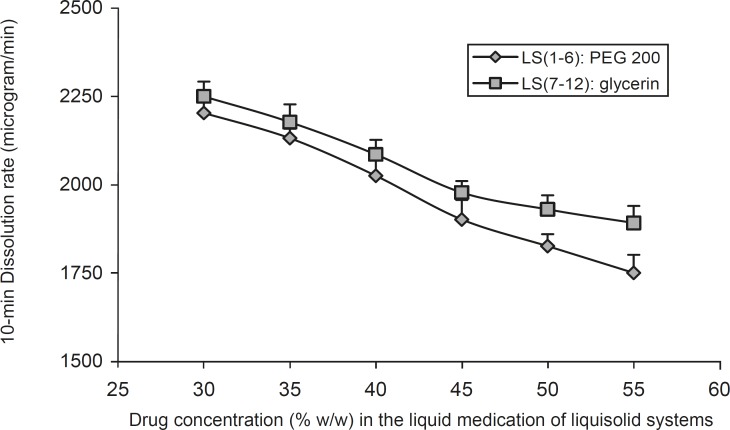
The effect of drug concentration (*C*_d_) in the liquid medication on the 10 min dissolution rate (*D*_R_) exhibited by different liquisolid formulations. Error bars are standard deviations for at least 4 determinations

Figure 3 shows the effect of the drug concentration (*C*_d_) in the liquid medication on the 10 min dissolution rate (*D*_R_) of indomethacin from the PEG 200 and glycerin liquisolid compacts. It can be seen that, as the concentration of the drug in the liquid medication (*C*_d_) decreased from 55% to 30% w/w, the values of *D*_R_ increased. The figure shows that the drug concentration in the liquid medication is one of the main factors on the performance of a liquisolid compact and has considerable effect on the indomethacin 10 min dissolution rate. It can be seen that *D*_R_ decreased with an increase in the concentration of liquid vehicle (PEG 200 or glycerin). However, when the concentration of indomethacin was increased from 50% to 55% in LS-2 and LS-1 formulations, there was no significant difference in *D*_R_ values (p = 0.27). In liquisolid tablets which containing glycerin, the increase of indomethacin’s concentration from 55% to 45% in LS-7 to LS-9, showed no significant differences in 10 min *D*_R_ values (p = 0.948 and p = 0.198 respectively). The same results were reported by Javadzadeh *et al*. (7); their study showed non significant increase in the dissolution rate, when the concentration of piroxicam was increased from 30% to 50% w/w. 

Such differences in the *D*_R_ values of indomethacin from liquisolid compacts observed in Figure 3 may be justified using the differences in the amount of soluble form of the drug or molecular dispersion states of the drug in the formulations. For instance, since the saturation solubility of indomethacin in PEG 200 is 8.93% w/w (Table 2), about 40.18% of the drug is as soluble form in LS-1 formulation (this formulation contains 55% drug and 45% PEG 200); whereas in LS-6 formulation, about 62.5% of indomethacin is as soluble form (LS-6 formulation contains 30% indomethacin and 70% PEG 200). In similar formulations (LS-7 and LS-12) which contains the same concentrations of drug and the same amount of glycerin, about 45.7% and 71.1% of indomethacin is as soluble form, respectively. 

**Figure 4:**
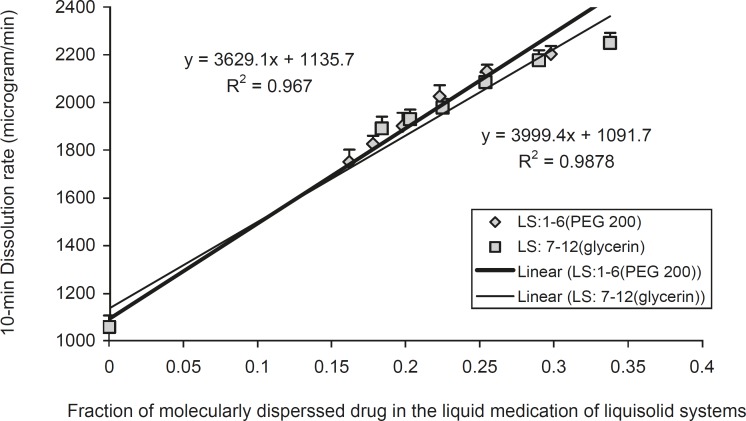
The effect of the fraction of molecularly dispersed drug (*F*_M_) in the liquisolid systems on the 10 min dissolution rate (*D*_R_) of indomethacin, exhibited by various liquisolid formulations. Error bars are standard deviations for at least 4 determinations

In order to investigate the effect of fraction of the dissolved or molecularly dispersed of indomethacin (*F*_M_) of the prepared liquisolid tablets on the dissolution rates of the drug from liquisolid compacts and DC tablets, the *F*_M_ was plotted against their corresponding *D*_R _values (Figure 4). 

F_M_ can be defined as the ratio of the drug’s saturation solubility (*C*_L_) in the liquid vehicle over the drug concentration (*C*_d_) in the liquid medication. Therefore, *F*_M_* = C*_L_*/C*_d_, where *F*_M _= 1 and when *C*_L_*/C*_d_ > 1. Based on the above equation, the *F*_M_ values of formulations are listed in Table 1 Since no liquid vehicle is involved in the case of directly compressed tablets which contain plain indomethacin powder, its *F*_M_ value was taken equal to 0. 

In Figure 4, the *D*_R_ in 10 min, increased in a linear manner, with increasing *F*_M_ values of the liquisolid systems can be seen. Therefore, it is possible to predict the dissolution rate (*D*_R_ in μg/min) of indomethacin liquisolid compacts containing PEG 200 or glycerin liquisolid tablets, which will be obtained within the initial 10 min of the dissolution process. A plot of *D*_R_ against *F*_M_ (when 0.162 < *F*_M _< 0.298 for PEG 200 contains liquisolid systems and when 0.184 < *F*_M_ < 0.338 for glycerin contains liquisolid formulations) shows that the *D*_R_ changes linearly with *F*_M_. The high correlation coefficients of 0.993 and 0.983 provide a value that characterizes the effect of *F*_M_ on indomethacin release from PEG 200 and glycerin liquisolid compacts, respectively. 

As shown in Figure 4, for systems with *F*_M_ values ranging 0.162-0.298 for PEG 200 containing liquisolid systems and when 0.184-0.338 for glycerin containing liquisolid formulations, the dissolution rate of indomethacin may be given by: 

*D*_R_ *= 3999.4 F*_M_ *+ 1091.7*

*(in PEG 200 containing liquisolid systems)*

*D*_R_ *= 3629.1 F*_M_ *+ 1135.7*

*(in glycerin containing liquisolid systems)*

**Figure 5:**
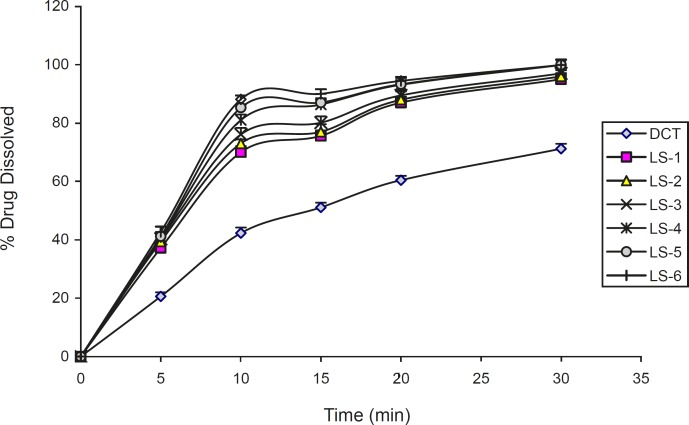
The dissolution profiles of indomethacin from liquisolid compacts containing different amounts of PEG 200. Error bars are standard deviations for at least 4 determinations

**Figure 6:**
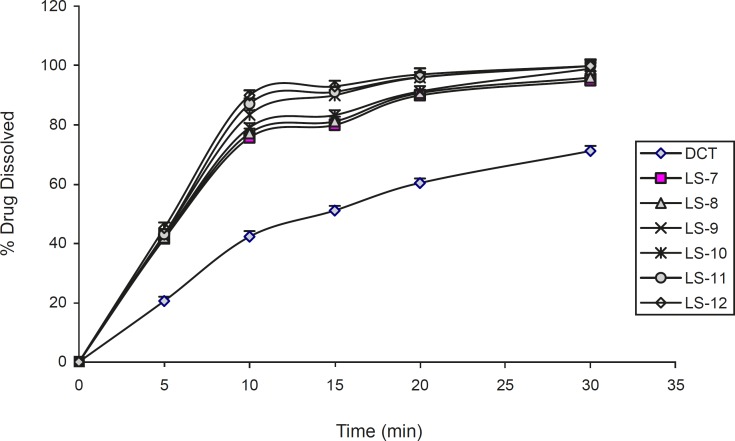
The dissolution profiles of indomethacin from liquisolid compacts containing different amounts of glycerin. Error bars are standard deviations for at least 4 determinations

The effect of the type and amount of vehicle on the release rate of indomethacin from liquisolid compacts is shown in Figures 5 and 6. It can be seen from the Figures, all two vehicles used in liquisolid compacts produced a higher dissolution rate than those of conventional tablet. The evaluation of the type liquid vehicle on release rate of investigated liquisolid formulations in LS-1 and LS-2 compared to LS-6 and LS-7 (with *C*_d_ of 55% and 50% w/w), showed a higher 10 min dissolution rate in liquisolid systems containing glycerin in comparison with those containing PEG 200 with similar amounts of liquid vehicle (p = 0.007). This difference was not observed after 20 min (p = 0.054 and p = 0.189, respectively). In other formulations with similar amounts of investigated liquid vehicles, no differences between dissolution rate after 10 and 20 min were observed (p > 0.05). The relationship between the percentage release of the drug and the solubility in medium was reported previously by Nokhodchi *et al. *(3). The similarity of the drug solubility in PEG 200 and glycerin caused small differences in drug dissolution rate in this investigated formulations. 

**Figure 7:**
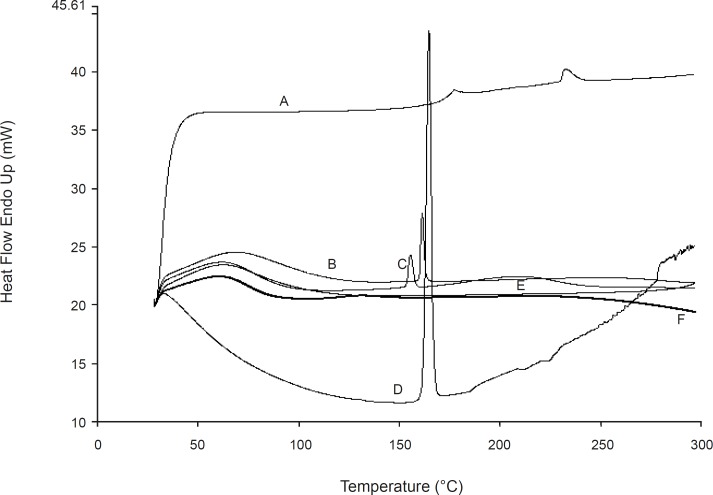
The differential scanning calorimetery of (A) silica, (B) physical mixture of directly compressed tablet, (C) LS-8 (containing glycerin), (D) indomethacin, (E) LS-2 (containing PEG 200) and (F) Avicel

DSC thermo grams are shown in Figure 7. Indomethacin showed an endothermic peak around its melting point. The liquisolid and physical mixture formulations showed the same peak in this area which indicates that there is no interaction between the drug and excipients or changes in crystallinity of the drug during the formulation process. From the above finding, it can be concluded that the enhanced dissolution rate of indomethacin liquisolid compacts is not due to the formation of a complex between the drug and excipients. 

## Conclusion

The liquisolid compacts technique can be a promising alternative for the formulation of water-insoluble drugs, such as indomethacin into rapid release tablets. The higher dissolution rates displayed by liquisolid compacts may also imply enhanced oral bioavailability due to the increased wetting properties and the surface of drug available for dissolution. It can also be concluded that from this study, the investigated liquisolid compacts of indomethacin, in different drug concentrations in their liquid medications, exhibits the drug dissolution rates which are directly proportional to the fraction, *F*_M_, of the molecularly dispersed drug in the liquid medication. 
